# Associations of Extrinsic and Intrinsic Components of Work Stress with Health: A Systematic Review of Evidence on the Effort-Reward Imbalance Model

**DOI:** 10.3390/ijerph13040432

**Published:** 2016-04-19

**Authors:** Johannes Siegrist, Jian Li

**Affiliations:** 1 Senior Professorship on Work Stress Research, Life Science Centre, University of Düsseldorf, Merowingerplatz 1a, Düsseldorf 40225, Germany; 2 Institute of Occupational and Social Medicine, Centre for Health and Society, Faculty of Medicine, University of Düsseldorf, Universitätsstrasse 1, Düsseldorf 40225, Germany; lijian1974@hotmail.com

**Keywords:** work stress models, effort-reward imbalance, over-commitment, systematic review, health measures

## Abstract

Mainstream psychological stress theory claims that it is important to include information on people’s ways of coping with work stress when assessing the impact of stressful psychosocial work environments on health. Yet, some widely used respective theoretical models focus exclusively on extrinsic factors. The model of effort-reward imbalance (ERI) differs from them as it explicitly combines information on extrinsic and intrinsic factors in studying workers’ health. As a growing number of studies used the ERI model in recent past, we conducted a systematic review of available evidence, with a special focus on the distinct contribution of its intrinsic component, the coping pattern “over-commitment”, towards explaining health. Moreover, we explore whether the interaction of intrinsic and extrinsic components exceeds the size of effects on health attributable to single components. Results based on 51 reports document an independent explanatory role of “over-commitment” in explaining workers’ health in a majority of studies. However, support in favour of the interaction hypothesis is limited and requires further exploration. In conclusion, the findings of this review support the usefulness of a work stress model that combines extrinsic and intrinsic components in terms of scientific explanation and of designing more comprehensive worksite stress prevention programs.

## 1. Introduction

Major changes in the nature of work and employment have occurred in many countries, particularly in high income ones, during the past several decades. The following are particularly noteworthy: first, employment sectors have shifted from industrial mass and lean production towards service delivery and information/communication technology-driven jobs; second, mainstream employment relations and job trajectories with long-standing continuity and security were increasingly replaced by more flexible arrangements, including mobility, retraining, de-standardization of employment contracts, and growth of job insecurity; third, with the advent of economic globalization, growing competition between transnational companies and the constraints of financial markets resulted in a sizeable increase of work pressure in many employment sectors, often in combination with decreasing job stability, in particular with the advent of the great financial crisis. In this context, levels of perceived work-related stress have been increasing over time in large parts of employed populations (for Europe see e.g., [1]). As a result, a growing burden of work stress-related poor health has been documented [2,3].

In view of the challenges of preserving working people’s health by reducing health-adverse levels of stressful work it is crucial to develop a common understanding of the underlying notion of stress at work, both at the conceptual and methodological level. Most experts agree that distinct demanding or threatening extrinsic features of job environments and employment conditions act as determinants of workers’ health, and many researchers claim that specific intrinsic characteristics (personality traits, coping behavior) increase the working people’s vulnerability to stress (e.g., [4,5]). Yet, there is no consensus on how this interaction is best defined and how extrinsic and intrinsic components are weighted with regard to their impact on health [6,7]. Some theoretical concepts of work stress focus exclusively on extrinsic features. The “demand-control model” represents probably the most prominent example of this line of research [8]. Other approaches maintain that individual factors play a significant role in shaping the stress process [9,10]. Accordingly, they accord primacy to individual-level approaches towards reducing stress at work, as evidenced in different reviews of stress intervention studies (e.g., [11,12]).

A third approach combines extrinsic and intrinsic factors within an integrative model. For instance, the “job demands-resources model” posits that personal resources as well as personal vulnerability factors influence working people’s wellbeing in addition to characteristics of the work environment. However, a clear-cut set of empirically tested hypotheses on how this interaction operates is currently still lacking [13]. “Effort-reward imbalance” is another example of a theoretical concept that combines extrinsic and intrinsic factors in studying work-related health [14]. With its focus on the work contract this model is based on the principle of social reciprocity where rewards received in return for efforts expended include money, esteem, and career opportunities (promotion, job security). The model asserts that lack of reciprocity (high effort in combination with low reward) generates strong negative emotions and stress responses with adverse long-term effects on health. A heightened susceptibility to these stress responses is expected among persons who exhibit a specific pattern of coping with demanding situations characterized by excessive engagement and a desire of being in control (“over-commitment”, OC).

The model’s hypotheses are as follows: (1) Each one of the three scales (“effort” and “reward” representing the extrinsic components, “over-commitment” representing the intrinsic component) exerts a direct effect on health; (2) The interaction of “effort” and “reward” (e.g., as ratio term quantifying the imbalance between high effort and low reward at individual level) exerts stronger effects on health that those observed in separate analyses; (3) The intrinsic component “over-commitment” moderates the effect of effort–reward imbalance on health, such that strongest effects are expected among people scoring high on “over-commitment”.

In this contribution we set out to review empirical evidence on the effort-reward imbalance (ERI) model in association with health, with a special focus on the intrinsic component and its moderating effect. This decision is based on the following considerations. First, to our knowledge, ERI is one of the few models representing this third approach to be tested in a variety of studies using different research designs. Therefore, its findings might help clarifying the debate about the relative importance of extrinsic *versus* intrinsic components of stressful work in association with health and the way of how their combined study can best be achieved. Second, in international research based on the ERI model, results on associations of the model’s extrinsic components with health have received far more attention than those dealing with its intrinsic component or with the interaction of these components. In part, this may be due to the fact that increased efforts and reduced rewards at work represent particularly stressful features of the significant changes in the nature of work and employment mentioned above. With their relevance for public health these features of the work environment may attract heightened attention among the research community [15]. Against this background a special emphasis on the model’s intrinsic component and its role as a moderator seems well justified. Third, two comprehensive systematic reviews on research analyzing the ERI model in its association with health have been published more than ten years ago [16,17]. Meanwhile, the body of empirical research has been growing rapidly, and an updated summary of this evidence has not been published.

### Objectives

Against this background we performed a systematic review of studies analyzing associations of effort-reward imbalance and over-commitment with health, with the following two objectives. First, we provide an overview of the distinct contribution of the model’s intrinsic component, over-commitment, towards explaining health, given a substantial respective body of recent evidence. By reviewing these findings, the relevance of extending the analysis of work stress beyond extrinsic factors is emphasized. Second, we review empirical support in favour of the third hypothesis mentioned above, *i.e.*, the moderation of associations of effort-reward imbalance with health by over-commitment. Testing this hypothesis has been neglected in many studies, and it is of interest to draw a critical balance on its validity.

## 2. Methods

### 2.1. Search Strategy

With this review, we followed the standard criteria of the PRISMA statement. We targeted studies published between January 1996 and December 2015 in English in peer-reviewed journals. This decision was based on the fact that the year of publication of the original international reference paper of the ERI model was 1996 [14]. Systematic search was undertaken in PubMed and PsycINFO (by Jian Li), using following search terms (“effort reward imbalance” or “effort-reward imbalance”) AND (“overcommitment” or “over-commitment”) identified in titles, abstracts and keywords. The literature search was also complemented by a vast collection of published papers on the ERI model (provided by Johannes Siegrist). Taken together, the search strategy yielded 314 records.

### 2.2. Inclusion and Exclusion Criteria

First, all 314 records were screened to check for duplicates, resulting in exclusion of 138 records. Second, we excluded papers that did not use psychometrically validated scales measuring “effort”, “reward” or “over-commitment”, either in the original version [18] or the more recently developed short version [19]. Third, we also excluded papers with findings based on cross-sectional studies that were limited to the analysis of main effects of effort, reward, and/or over-commitment, and that used exclusively self-report data. Due to the problem of common method variance in associations between stressful work and health respective publications add little to the available knowledge.

However, keeping in mind our first objective, we included findings from cross-sectional studies in our review in addition to those based on longitudinal, or experimental (defined as additional stress test or neuroendocrine test performed in laboratory) study designs if associations with validated biomedical health data were reported. Twenty-seven papers met these selection criteria for the first objective.

To explore our second objective, we included all studies, whether cross-sectional, longitudinal or experimental, that tested the interaction of intrinsic with extrinsic model components. To this end, studies reporting results based on the established statistical test of a moderation effect (ERI × OC) were included (21 studies). Additionally, given the fact that several epidemiological studies instead analysed associations of a single 3- or 4 categorical variable offering combinations of interest between the extrinsic and intrinsic component (e.g., ERI+OC+, ERI-OC+, ERI-OC+, ERI-OC-) with health, we included these latter studies (6 studies). Here, it was of interest to explore to what extent associations with health were stronger in the high stress group (ERI+OC+) *vs.* remaining groups. Totally, 27 papers were included for the second objective.

**Figure 1:**
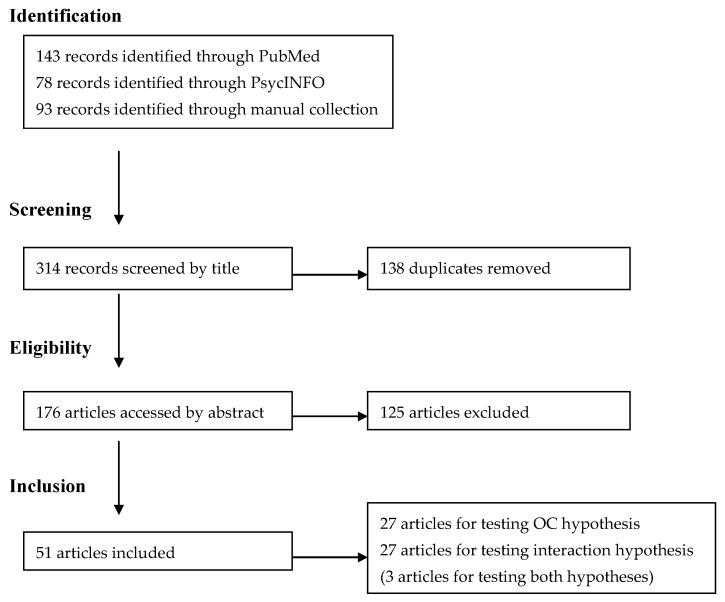
Flowchart of included and excluded articles.

By applying these selection criteria, a total of 54 reports were identified as the basis of this review. It turned out that three reports provided information on both study objectives. Thus, the number of publications included was 51 (see Figure 1). Our evaluation was based on a careful assessment of information provided in the Abstracts. Additionally, original papers were consulted in case of uncertainty, and joint decisions based on independent judgments of both authors were made in these cases.

### 2.3. Analysis

To standardize data collection information on study characteristics, relevant variables and associations was computed in standardized form, including author, study design, country, sample size, study population, health outcome, and classification of main findings. To synthesize evidence, the findings were classified according to the two objectives of the review (objective 1: Table 1; objective 2: Table 2), and tables were further subdivided according to overarching topics. Instead of presenting detailed empirical study findings we indicated whether statistically significant associations according to the hypotheses tested were observed or not, and we indicated the direction of significant associations (positive, negative). We considered all results with a *p* value less than 0.05 to be statistically significant.

Due to the heterogeneity of study designs and statistical analyses, a meta-analysis was not a feasible option, instead the results from included studies were combined into a narrative synthesis. Narrative synthesis is a method used to synthesis results from multiple studies, which use a range of methods in order to draw conclusion.

## 3. Results

### 3.1. Objective 1: Over-Commitment and Health

The findings addressing the first objective of this review, *i.e.*, exploring the contribution of over-commitment towards explaining health, are displayed in Table 1. In the upper part, associations of over-commitment with either specific diseases or with their major biomedical or behavioral risk factors are indicated. Studies are presented according to their design (prospective/longitudinal: five reports [20,21,22,23,24] *vs.* cross-sectional/case-control: seven reports [25,26,27,28,29,30,31]). In the lower part of the table, associations of over-commitment with physiological variables are displayed. These variables are used as markers of potential psychobiological pathways mediating stressful experience with disease development. They include studies with experimental or cross-sectional design, where cardiovascular (six reports) [32,33,34,35,36,37], immune (three reports) [38,39,40] and hormonal parameters (seven reports) [32,41,42,43,44,45,46] were analyzed. As one report analyzed two different psychobiological markers [32] the number of studies presented in Table 1 is 27 instead of 28.

In four out of five studies based on a prospective/ longitudinal design over-commitment was associated with the outcome of interest, *i.e.,* a high level of over-commitment resulted in an increase of systolic blood pressure over time [20], in an elevated risk of fatigue [21] and of insomnia [22], and in a higher rate of restenosis of coronary artery in cardiac patients [23]. However, no effect of this intrinsic component on smoking cessation over time was observed [24]. Concerning cross-sectional and case-control studies, six out of seven studies found that over-commitment was positively associated with elevated risks of dyspepsia [25], high glucose [26], high fibrinogen [27], dyslipidemia [28], coronary stenosis [29], and an increased carotid intima media thickness (among women only) [30]. However, over-commitment was not associated with increased risk of depression [31]. Rather, this study found a strong association with the model’s extrinsic component.

Results in the lower part of Table 1 relate to physiological measures. Ambulatory blood pressure over the working day was increased among over-committed employees in one study, in combination with low occupational position [32], whereas ambulatory heart rate over the working day was increased in association with ERI, but not with OC in another study [33]. In this latter study an association of OC with increased basal sympathetic drive was observed [34]. A recent report observed significant effects of both components, ERI and OC, on reduced heart rate variability [35]. Two further publications introduced fibrinolysis and blood coagulation respectively as potential stress-related biological markers [36,37]. In both cases, OC but not ERI was associated with impaired functioning.

Immune system-related variables analyzed in this context include natural killer (NK) cells, T helper cells, and C-reactive protein (CRP) as an inflammatory marker. Two reports found significant associations of OC with increased CRP [38] and with reduced NK cells respectively. In this latter study, a lower increase of T helper cells following stress was also observed in association with OC and to some extent with ERI [39]. In a Japanese study on men and women, a robust relationship of ERI, but not OC, with reduced number of NK cells was observed among men, whereas this tendency among women was not statistically significant [40]. Seven reports studied associations of components of the ERI model with the stress hormone cortisol (in two studies the associated hormone ACTH). Findings are inconsistent, in part due to different measures (awakening response *vs.* diurnal pattern; salivary assessment *vs.* blood samples) and different designs (experimental *vs.* naturalistic). In 4 out of 7 studies, some evidence of increased cortisol secretion was found in association with OC, more so for men than for women [32,41,42,43]. In one of these studies [41] OC was associated with elevated awakening cortisol, but also with lower cortisol concentrations in the afternoon till bedtime. Another study testing the responsiveness of this stress hormone axis observed a reduced release of ACTH and cortisol in association with OC [44]. A further study reported a reduced cortisol awakening response associated with ERI, but not with OC [45], whereas in one study no association of cortisol excretion with the ERI variables was found [46].

The results of this part of the review are best summarized as follows: first, a majority of reports document associations of over-commitment, the intrinsic component of the ERI model, with the health indicators under study. This information supports the notion that OC exerts independent, statistically and clinically relevant effects on different health indicators under study (thus supporting hypothesis 1 stated above in 10 out of 12 studies). Second, a majority of reports studying psychobiological markers of stress found significant associations with over-commitment (11 *vs.* four studies). However, the direction of associations was not always consistent, in particular in studies using cortisol as a marker. Here, positive and negative associations were observed, and both directions can be reconciled with the current state of research (see Discussion). Preliminary evidence that excessive striving at work contributes to endogenous inflammation, reduced immune competence, impaired blood coagulation and fibrinolysis calls for further investigations along these lines.

### 3.2. Objective 2: Test of Interaction

As a second objective of this review we set out to explore to what extent the model’s interaction hypothesis has received empirical support. The findings of 27 studies are summarized in Table 2. Three of these studies provided additional data included in Table 1 [20,37,41]. In the main part, studies are listed that performed the conventional statistical interaction test ERI × OC. Twenty-one reports were identified to this end [20,37,41,47,48,49,50,51,52,53,54,55,56,57,58,59,60,61,62,63,64]. As explained in the Methods section, studies are listed in the lower part of the Table that analyzed the different strength of associations with health of a composite variable that combined data from the model’s extrinsic and intrinsic components (6 studies [65,66,67,68,69,70]). Clearly, this approach which is more convenient in epidemiological than psychological studies does not comply with the statistical requirements testing a moderator effect, but provides some preliminary evidence along these lines.

Obviously, a majority of studies failed to support the moderation hypothesis of over-commitment. In 13 reports a respective statistical test was not significant [20,37,41,47,48,49,50,51,52,53,54,55,56]. On the other hand, eight studies reported a significant interaction term [57,58,59,60,61,62,63,64]. With one exception [57] they are based on a cross-sectional or case-control study design, where five include a self-report subjective measure and two a biomedical objective measure (hypertension; coronary heart disease) [58,59]. Li *et al.* [60] report a significant synergy index (indicating synergistic interaction) with an odds ratio of 6.7 of poor mental health resulting from the interaction of ERI and OC. A significant synergy effect is also reported in a longitudinal study where a significantly increased risk of poor physical functioning results from the interaction of ERI and OC. However, such an effect is not observed for poor mental functioning [57]. In a study from China, a significant synergy index is reported for the interaction of ERI and OC with risk of hypertension, where an almost three times elevated prevalence odds ratio is observed in the high risk group [58]. In an earlier study this interaction term resulted in a significantly elevated prevalence odds ratio of documented coronary heart disease in a case-control study [59]. Feuerhahn *et al.* [61] and Bakker *et al.* [62] find support of the moderation hypothesis with regard to measures of burnout, and the same holds true for depressive symptoms [63] and organizational well-being such as job satisfaction and turnover intention [64].

Concerning the studies that constructed a combined measure of extrinsic and intrinsic components all reported elevated odds ratios for the high stress *vs.* low stress group in logistic regression analyses, where the high stress groups were defined by a co-manifestation of high effort-reward imbalance and a high level of over-commitment. This was particularly impressive in a longitudinal study on mental health among young physicians [65], and in a large study of poor mental health among female nursing home staff [66]. Four further studies provide findings along these lines [67,68,69,70].

In summary, the moderation hypothesis (hypothesis 3 mentioned above) is not confirmed in a majority of studies. However, about one third of the studies (eight out of 21) reported results in favor of this hypothesis, and a statistically less conclusive approach based on a composite measure combining data on extrinsic and intrinsic components supported the notion that the impact of high effort and low reward on health is particularly adverse among people who are over-committed to their work.

## 4. Discussion

After more than 10 years, this is the first comprehensive review of results from studies on associations of work stress in terms of effort-reward imbalance with health, with a specific focus on the model’s intrinsic component over-commitment and the related moderation hypothesis. According to our selection criteria 51 reports were retrieved from a total of 176 papers published between 1996 and 2015. Twenty-seven reports provided findings exploring associations of over-commitment with a variety of health indicators, and main results were summarized in Table 1. Twenty-seven reports were identified that examined the interaction of over-commitment and effort-reward imbalance in associations with health, with an overview of results displayed in Table 2. Three of these studies also provided information included in Table 1 [20,37,41].

People who are over-committed to their work are more likely to exhibit major cardiovascular risk factors, such as high blood pressure, atherogenic lipids, high level of glucose and fibrinogen, or sub-clinical manifestations of cardiovascular disease. Their pro-inflammatory activity is increased and their immune competence in terms of natural killer cells is reduced. They suffer more often from fatigue and insomnia than their more relaxed, less involved colleagues. Findings are in line with pathogenic consequences of a chronically aroused sympatho-adrenergic system of the organism that remains unbalanced by restoring recovery processes of the vagal component of the autonomic nervous system [71]. Although a majority of studies included in this part of the review provide significant findings a few reports failed to observe a link between over-commitment and health. Moreover, considerable inconsistency was obvious from experimental or naturalistic studies monitoring stress hormones, such as cortisol or ACTH, where some studies reported an increased hormonal release and some a reduced responsiveness.

In addition to an explanation related to differences in measurement, design, or study samples this inconsistency of responsiveness of stress hormones in association with work stress measures can be explained in the frame of a proposed two-stage model of stress reactivity over the life course [72]. According to this model, in early stages of a person’s career of exposure to stressful working conditions, the stress axes within the organism are expected to respond with excessive drive, resulting in a high release of stress hormones, whereas in the long run, reduced release and dampened responsiveness could be the result of a functional adaptation to recurrent excessive exposure-related activation. This body of knowledge about health-adverse effects of over-commitment is complemented by a substantial amount of related findings based on the extrinsic components of the effort-reward imbalance model. These latter publications were not included in this analysis, but an updated review of empirical evidence of the full model is currently in press [15].

As a second objective of this contribution, we collected data on the moderation hypothesis from 27 reports. Of the 21 studies testing the hypothesis by an interaction term ERI × OC, 13 failed to support it, while eight reported significant results in its favor. More positive findings were observed if analyses were based on the construction of a variable that combines information on extrinsic and intrinsic components (all six studies with positive results) although this approach is not in line with the established statistical way of testing interaction. It is obvious that the third one of the three core hypotheses of the ERI model mentioned above (moderation) has received far less robust empirical support so far than is the case for the hypothesis of a direct effect of over-commitment on health. Although confirmatory factor analyses of the scales measuring the model repeatedly demonstrated that over-commitment results as a distinct uni-dimensional factor, substantial correlations of its scale with the scales “effort” and “reward” were observed [18,19]. We cannot exclude that these correlations reduce the probability of observing a statistically significant moderation effect. In view of the fact that eight reports observed a moderation effect of over-commitment in the expected direction, and given additional preliminary evidence from studies using a composite measure of extrinsic and intrinsic data, it is concluded that this hypothesis deserves further analysis in future studies.

To our knowledge no alternative theoretical model of stressful work addressing extrinsic and intrinsic components at the conceptual and measurement level has been investigated as extensively as is the case with the ERI model. The findings summarized in this review are derived from different study designs and different population groups, and they represent a wide spectrum of health measures assessed by self-report and objective biomedical data. Results lend support to the notion that a “black box” approach towards assessing stressful work that bypasses individual coping dimensions falls short of representing the full range of health-adverse consequences. This conclusion has direct implications for the design of prevention and health promotion strategies at the workplace as it suggests increased health benefits of programs that combine structural, organization-based measures with measures of improved personal coping with stress at work [73]. A second strength of this contribution concerns the comprehensive feature of the review. Based on the search criteria we identified 176 publications by combining web based and manual search information and by covering a reporting period of the past 20 years. We excluded studies with relatively weak scientific quality (cross-sectional studies testing main effects only and using self-report data for assessing work stress and health) and studies based on proxy measures instead of the psychometrically validated scales of the model.

### Limitations

Despite these merits this review suffers from several limitations. First, with our search terms and selection criteria we may have bypassed some relevant publications. Equally, we run the risk of missing relevant contributions from book chapters and other publication formats not included in PubMed or PsycINFO, and, although less likely, from additional databases. Second, given the diversity of study designs, health measures, and statistical procedures of data analysis the information provided in the tables suffers from a lack of comparability and precision, thus preventing a meta-analytic approach to the topic under study. Third, as this review largely focused on over-commitment, we were not able to perform a systematic comparison of the relative explanatory contribution of its extrinsic *vs.* intrinsic components. Such a comprehensive, obviously much more extensive review is considered an important step of future research. Fourth, we only included journal articles in English. Research findings written in other languages were not well considered in this current review. Fifth, the findings resulting from this review are likely to be influenced by publication bias. As is the case with other subjects, publication of positive findings is more likely than publication of negative results. We were not in a position to evaluate the potential extent of this bias. Moreover, although we adhered to the quality criteria of conducting systematic reviews a potential reporting bias cannot be excluded as both authors are involved in scientific research on the ERI model since many years.

Finally, this review suffers from its rather narrow scope as it focuses exclusively on one theoretical model of work stress. Meanwhile, a number of publications provide results from analyses that combine the ERI model with the demand-control or the organizational injustice model. As these models complement each other, such extensions are well justified. The same limitation concerns the model’s intrinsic component. Several suggestions were made to expand the range of intrinsic factors that might act as modifiers of associations of effort-reward imbalance with health, both in a mitigating and in an aggravating way. Examples include the assessment of reward sensitivity [52], of coping strategies of selection, optimization, and compensation [74], or of recovery resources [75].

## 5. Conclusions

This review has demonstrated robust evidence of associations of an intrinsic component of work stress, the individual coping pattern of over-commitment to work, with a variety of health indicators. These findings suggest that highly overcommitted people are at elevated risk of suffering from adverse health in the long run. Limited support only was found in favor of a potential role of over-commitment as moderator of relationships between effort-reward imbalance and health. While this review identified gaps of knowledge that define challenges for further research it nevertheless provides a strong argument in favor of combining structural, organization-based measures with tailored person-based approaches in programs of primary and secondary prevention tackling the challenges of health-adverse stressful work.

## Figures and Tables

**Table 1:** Summary of effects of over-commitment on health.

Authors [Reference]	Study Design	Sample	Health Outcomes	OC Hypothesis
Diseases or risk factors				
Gilbert-Ouimet *et al.* [20]	P	1595 Canadian white-collar workers	Blood pressure	↑
Sembajwe *et al.* [21]	P	14,641 French company employees	Fatigue	↑
Ota *et al.* [22]	P	1022 Japanese workers	Insomnia	↑
Joksimovic *et al.* [23]	P	106 German workers with coronary artery disease	Coronary restenosis	↑
Ota *et al.* [24]	P	579 Japanese workers	Smoking cessation	-
Rothenbacher *et al.* [25]	CS	189 German company employees	Dyspepsia	↑
Irie *et al.* [26]	CS	441 Japanese workers	Plasma glucose	↑
Xu *et al.* [27]	CS	732 Chinese workers	Plasma fibrinogen	↑
Xu *et al.* [28]	CS	544 Chinese workers	Dyslipidemia	↑
Xu *et al.* [29]	CS	320 Chinese workers	Coronary stenosis	↑
Xu *et al.* [30]	CS	734 Chinese workers	Carotid intima-media thickness	↑
Lehr *et al.* [31]	CC	244 German teachers	Depression	-
Psychobiological markers				
Steptoe *et al.* [32]	CS	197 British white-collar employees	Ambulatory blood pressure, and salivary cortisol	↑ (ambulatory blood pressure, salivary cortisol)
Vrijkotte *et al.* [33]	CS	109 Dutch white-collar workers	Ambulatory heart rate	-
Vrijkotte *et al.* [34]	CS	67 Dutch white-collar workers	Ambulatory cardiac sympathetic activity	↑
Garza *et al.* [35]	CS	91 Dutch office workers	Heart rate variability	↓
Vrijkotte *et al.* [36]	CS	124 Dutch white-collar workers	Plasma fibrinolysis	↓
von Känel *et al.* [37]	E	52 German teachers	Plasma coagulation	↓
Xu *et al.* [38]	CS	731 Chinese workers	C-reactive protein	↑
Bellingrath *et al.* [39]	E	55 German teachers	Natural killer cells and T-helper cells	↓ (natural killer cells and T-helper cells)
Nakata *et al.* [40]	CS	347 Japanese white-collar employee	Natural killer cells	-
Marchand *et al.* [41]	CS	401 Canadian workers	Salivary cortisol	↑ (awakening) ↓ (afternoon and bedtime)
Wirtz *et al.* [42]	E	200 German employees	Plasma cortisol	↑
Eller *et al.* [43]	CS	83 Danish workers	Salivary cortisol	↑
Bellingrath *et al.* [44]	E	53 German teachers	Plasma adrenocorticotropin, plasma and salivary cortisol	↓ (plasma adrenocorticotropin, plasma and salivary cortisol)
Maina *et al.* [45]	CS	104 Italian workers	Salivary cortisol	-
Ota *et al.* [46]	CS	115 Japanese teachers	Salivary cortisol, and dehydroepi-androsterone	-

P: prospective study; CS: cross-sectional study; CC: case-control study; E: experimental study; ↑: significant and positive association; ↓: significant and negative association; -: non-significant or null association.

**Table 2:** Summary of moderating effects of over-commitment on associations between effort-reward imbalance and health.

Authors [Reference]	Study Design	Sample	Health Outcomes	Interaction Hypothesis
Test of interaction term (ERI × OC)				
Gilbert-Ouimet *et al.* [20]	P	1595 Canadian white-collar workers	Blood pressure	-
von Känel *et al.* [37]	E	52 German teachers	Plasma coagulation	-
Marchand *et al.* [41]	CS	401 Canadian workers	Salivary cortisol	-
Bathman *et al.* [47]	CS	66 Australian dairy farmers	Salivary immunoglobulin A	-
Oren *et al.* [48]	CS	159 Israeli employees	Burnout	-
Yu *et al.* [49]	CS	878 Chinese factory workers	Psychosomatic complaints, and depressive symptoms	-
van Vegchel *et al.* [50]	CS	167 Dutch healthcare workers	psychosomatic and physical health symptoms	-
Preckel *et al.* [51]	CS	1587 German industrial workers	Exhaustion, depression, and sleep	-
Allisey *et al.* [52]	CS	897 Australian police officers	Psychological distress	-
Derycke *et al.* [53]	P	1531 Belgian healthcare workers	Turnover intention	-
Willis *et al.* [54]	CS	112 British police employees	Work-family conflict and burnout	-
Tse *et al.* [55]	CS	186 British bus drivers	Psychological and physical ill health	-
Aboa-Éboulé *et al.* [56]	P	738 Canadian post-myocardial infarction workers	Recurrent coronary events	-
Wahrendorf *et al.* [57]	P	6053 French company employees	Physical health functioning	+
Xu *et al.* [58]	CS	734 Chinese workers	Hypertension	+
Xu *et al.* [59]	CC	388 Chinese workers	Coronary heart disease	+
Li *et al.* [60]	CS	2738 German industrial workers	Mental health functioning	+
Feuerhahn *et al.* [61]	CS	152 German company employees	Emotional exhaustion and job performance	+
Bakker *et al.* [62]	CS	204 German nurses	Burnout	+
Jolivet *et al.* [63]	CS	3316 French nurses	depressive symptoms	+
Kinman *et al.* [64]	CS	844 British academic employees	Job satisfaction and turnover intention	+
Test of combined variable of ERI and OC				
Buddeberg-Fischer *et al.* [65]	P	433 Swiss physicians	Anxiety and depression	+
Pélissier *et al.* [66]	CS	2471 French nursing home staff	Mental well-being	+
Yu *et al.* [67]	CS	5338 Chinese workers	Depressive symptoms	+
Lau [68]	CS	1803 Norwegian employees	Self-rated health, and burnout	+
Feldt *et al.* [69]	P	298 Finnish managers	Burnout, and recovery experiences	+
Weyers *et al.* [70]	CS	367 Danish nurses	Health functioning, and somatic symptoms	+

P: prospective study; CS: cross-sectional study; CC: case-control study; E: experimental study; +: significant interaction; –: non-significant interaction.
